# Stroke in young adults in the Middle East and North Africa region: What is the difference from elsewhere? A report from sixteen centers experiences

**DOI:** 10.3389/fneur.2025.1653599

**Published:** 2026-04-10

**Authors:** Amal M. Al Hashmi, Yahia Imam, Mehdi Farhoudi, Elyar Sadeghi, Andoerza Ghoreishi, Abdulkarim Al Mutairi, Khaled Mohamed Sob, Atilla Ozcan Ozdemir, Erdem Gurkas, Hani Humaidan, Hosam Al-Jehani, Faiza Mushtaque, Vinitha Leeleamani, Salma Said, Hany Helal, Noora Al Jahwari, Abeer Sabry Safan, Rasha K. M. Abumustafa, May Al Hamid, Farid Aladham, Ossama Yassin Mansour, Haytham Hussein Osman, Sachin Jose, Ehab Shawky

**Affiliations:** 1 Khoula Hospital, Ministry of Health of Oman, Muscat, Oman; 2 Head Neuroscience Center, Oman International Hospital (OIH), Muscat, Oman; 3 Oman Stroke Society, Tabriz, Iran; 4 Neuroscience Institute, Hamad Medical Corporation, Doha, Qatar; 5 Neuroscience Research Center, Tabriz University, Tabriz, Iran; 6 School of Medicine, Vali-e-Asr Hospital, Zanjan University of Medical Science, Zanjan, Iran; 7 Prince Sultan Hospital Military Medical City, Riyadh, Saudi Arabia; 8 Department of Neurology, Al Hussain Hospital, Al Azhar University, Cairo, Egypt; 9 Neurology Department, Eskisehir Osmangazi University, Istanbul, Türkiye; 10 Latfi Hospital, Istanbul, Türkiye; 11 Salmaniya Medical Complex, Al Damam, Bahrain; 12 Department of Neurosurgery, Interventional Neuroradiology, Imam Abdurrahman Alfaisal University, Alexandria, Saudi Arabia; 13 Department of Neurology and Neurosurgery, Montreal Neurological Institute and Hospital, McGill University, Montreal, QC, Canada; 14 Department of Neurosurgery, Houston Methodist Hospital, Weill Cornell University, Houston, TX, United States; 15 Department of Medicine, Sohar Hospital, Sohar, Oman; 16 Department of Neurology, Faculty of Medicine, Alexandria University, Al Khobar, Egypt; 17 Tanta University Hospital, Tanta, Egypt; 18 Ministry of Health of Oman, Muscat, Oman; 19 Department of Neurology, King Fahad Hospital of the University, Muscat, Saudi Arabia; 20 Specialty Hospital, Amman, Jordan; 21 Neurology Department, The National Ribat University, Khartoum, Sudan; 22 Oman Medical Specialty Board (OMSB), Muscat, Oman

**Keywords:** stroke, stroke subtypes, young adults, risk factors, etiology, MENA region

## Abstract

**Background:**

Stroke at a young age is on the rise globally. The diverse range of underlying etiologies and risk factors for stroke in young adults make it challenging. There are very limited papers in this regard from our Middle East and North Africa (MENA) region.

**Objectives:**

Identifying risk factors, etiologies and treatment offered to young stroke patients in the MENA region.

**Method:**

A five-year multicenter retrospective study across nine MENA countries (2018–2022) was conducted. Multinomial logistic regression was applied to evaluate associations between vascular risk factors and stroke subtypes (adjusted for age, sex, and country).

**Results:**

16 centers participated including 3,971 patients, 65.5% were male. Hypertension (HTN) was the most common risk factor among all stroke subtypes (38%). Diabetes mellitus (DM) and HTN, were the most frequent risk factors for ischemic stroke (IS) and Intracerebral hemorrhage (ICH). Smoking and HTN were common in subarachnoid hemorrhage (SAH). All patients had either Cerebral Computed topography (CT) angiogram or Carotid Doppler, 32.6% of 67.4% showed positive results. A total of 2,441 (61.9%) patients had Transthoracic Echocardiogram (TTE) and/or Transesophageal Echocardiogram (TEE). 11.2% of the patients showed abnormalities. The antiphospholipid screening tests were done in 31.4% of the patients and were positive only in 3.5%. IS was the most common stroke subtype 63.6%, followed by (ICH) 19.2%. Undetermined etiology (UDE) was the most common underlying etiology of IS in 22.2%. Intravenous thrombolysis therapy (IVT) was given in 8.5% of patients. Mechanical thrombectomy (MT) was performed in 3.7%. Anticoagulation therapy was offered in 20% of patients. Endovascular treatment (EVT) was performed in 5.2% of patients. Surgical intervention including clipping or hemicortectomy or hematoma evacuation was done in 5.6% of patients.

**Conclusion:**

This study offers the first regionally coordinated effort to examine the risk factors, and etiologies of stroke in young adults across the (MENA) region. The data clearly reflects not only the high burden of disease but also striking variability in patient profiles and healthcare responses between countries. Emerging non-traditional risk factors, and persistent diagnostic limitations, point to the complexity of tackling stroke in the young. Yet, in this complexity lies opportunity.

## Introduction

In recent years, the global age of stroke has declined, largely due to a rising incidence among younger adults (1). While stroke care has seen remarkable advances over the past two decades, stroke in the young remains a complex and often under recognized entity. Typically defined as occurring before age 45 or 50, young adult stroke presents distinct epidemiological features, risk factors, and management challenges across regions (2, 3).

In the Middle East and North Africa (MENA), understanding stroke in young adults is particularly difficult. The region’s wide geographical span, diverse public health profiles, and uneven healthcare infrastructure hinder unified insights (4). Many health systems in the MENA region lack the capacity for robust data collection and longitudinal reporting (5). This makes it challenging to grasp the full scope of stroke—especially given its multifaceted phases (hyper -acute, acute, subacute, and chronic), each requiring tailored strategies across the care continuum (6, 7).

Although stroke is typically associated with older age, more cases are now being seen in younger patients during routine clinical encounters. These cases often lack traditional vascular risk factors or involve overlapping causes, complicating diagnosis and care. This complexity is amplified in the MENA region, where limited data and variable care pathways obscure the unique clinical and etiological landscape. Known contributors to young-onset stroke include sickle cell disease, Moyamoya disease, genetic syndromes, and cardiac anomalies such as patent foramen ovale (8). However, regional data on these trends remain scarce (9).

Against this backdrop, our study aims to examine the burden of stroke in young adults across MENA, with a focus on risk factors, stroke subtypes, underlying etiologies, and treatment practices.

## Objectives

This study aims to identify risk factors, stroke subtypes, etiologies and general reporting on therapies offered to stroke patients in the MENA region.

## Methods

This is a 5-year (2018–2022) retrospective multicenter study performed in the (MENA) region. Vascular neurologists/ neuro-interventionists in the MENA region were contacted through a Zoom meeting to provide information on young stroke patients admitted acutely at their hospitals. There was a total of sixteen participating centers from the following nine countries Bahrain, Egypt, Iran, Jordan, Oman, Saudi Arabia, Sudan, Qatar and Turkey.

Patient’s data was retrieved from electronic medical records system. Data was collected in data sheets, and a confidential code was assigned to each patient. No personal data was divulged in the data collection sheets except age and sex. All the data was treated with confidentiality. Acute stroke was defined as sudden onset neurologic deficit due to a vascular etiology. All stroke subtypes were included ischemic stroke (IS), intracerebral hemorrhage (ICH), cerebral sinus venous thrombosis (CSVT) and subarachnoid hemorrhage (SAH). Detailed history, examination, and brain imaging (CT or MRI) were used to confirm the diagnoses. Mimics and transient ischemic attacks (TIAs) were excluded. We considered rare etiologies if diagnostic tests confirmed clotting abnormalities in cases where no other etiology was apparent. A diagnosis of IS related to patent foremen ovale (PFO) with or without atrial septal aneurysm (ASA) or dissection was considered in patients where no other etiologies explained the symptoms and imaging was suggestive. Vasculitis screens including antinuclear antibodies (ANA), anti-phospholipid antibodies rheumatoid screen, thrombophilia screen, homocysteine and sickling were done in some patients when needed. Abnormalities revealed by the above tested are considered as an etiology in case no other explanation was found. Traumatic or iatrogenic etiologies were excluded for all the stroke subtypes. Trial of Org 10,172 in Acute Stroke Treatment classification (TOAST) classification was performed locally by certified vascular neurologists following a unified data dictionary distributed to all participating centers. Each country implemented peer validation by a second investigator, and outlier distributions were re-audited against raw data.

### Patient description

Patients aged 18–45 years with a definite diagnosis of acute stroke were included. Both sexes were included. All patients were admitted under the supervision of vascular neurologists/interventionists at stroke units with the availability of appropriate diagnostic tools and monitoring farcicalities. All patients had routine blood tests, a chest x-ray, an electrocardiogram (ECG) and brain imaging on admission. All patients had confirmation of acute stroke by neuroimaging Head computed tomography (CT) or Magnetic Resonance Imaging (MRI). Carotid Doppler or cerebral CT angiogram were done in all patients. Nearly all patients with acute ischemic stroke had a transthoracic echocardiogram (TTE) and or trans-esophageal echocardiogram (TEE) requested. Magnetic resonance venography (MRV) or CT Venography (CTV) were performed to confirm the diagnosis of CSVT. Additional investigations such as conventional cerebral angiograms were carried out in patients where the etiology was not clear following the completion of the initial first-line investigations to help identify rare etiologies. CTA or carotid Doppler was considered positive if it demonstrated ≥50% luminal stenosis, or if any vascular abnormality was identified, including aneurysm, dissection, basilar stenosis, or other structural lesions (e.g., non-significant stenosis <50%, carotid web, atherosclerotic plaque, or diffuse wall thickening).

### Statistical analysis

Continuous variables were presented as mean and standard deviation, whereas categorical variables were presented as frequency and percentage. A comparison of means between two independent groups was carried out using the independent samples *t*-test. The association between two categorical variables were tested using a Chi-square test (Fisher’s exact/Likelihood ratio). In addition, multinomial logistic regression was used to examine associations between vascular risk factors (HTN, DM, dyslipidemia (DLP), smoking, Atrial fibrillation (AF), Ischemic Heart Disease (IHD), and other risk factors) and stroke subtypes (IS, ICH, SAH, CVST), as well as TOAST categories for ischemic stroke. A complete-case approach was applied for missing data (<5% overall). Multicollinearity was assessed via variance inflation factors (VIF), with all values <2. For multinomial logistic models, Ischemic Stroke (IS) served as the reference category for stroke-subtype comparisons, and Large Vessel Disease (LVD) served as the reference for TOAST analyses. Model fit was verified using likelihood-ratio and goodness-of-fit tests, and residual diagnostics were reviewed for influential points. All models were adjusted for age, sex, and country. Detailed outputs are provided in (Supplementary File).

Results are expressed as adjusted odds ratios (aOR) with 95% confidence intervals. Country was included as a block effect to account for between-country heterogeneity.

All the analysis was carried out in IBM SPSS statistics version 30.

### Ethical consideration

Ethical approval was obtained from the Ministry of Health of Oman (MOH/OSH/REC/21/2022), which served as the primary oversight body as the anonymized dataset was stored and analyzed in Oman. Each site investigator was responsible for securing the appropriate ethical approval or exemption from their respective institutional review board or ethics committee in accordance with local regulations and all patient data were fully anonymized before transfer and analysis.

## Results

### Demographic data, risk factors and stroke subtypes

A total of 3,971 patients from 16 centers were included. Country representation was heterogeneous, dominated by Qatar (29%) and Iran (23%) among which 65.5% were male, with a mean age of 37.66 ± 6.77 years (Table 1).

**Table 1:** Socio demographics, risk factors, stroke types, investigations and treatments.

Variable	*n* (%)
Sex (*N* = 3,971)	
Female	1,369 (34.5)
Male	2,602 (65.5)
Age (mean ± SD)	37.65 ± 6.77
Age (median Q1–Q3)	39 (34–43)
Country	
Qatar	1,157 (29.1)
Iran	901 (22.7)
Egypt	554 (14.0)
Turkey	516 (13.0)
Oman	482 (12.1)
Saudi Arabia	218 (5.5)
Bahrain	121 (3.0)
Others	22 (0.6)
Risk factors	
Hypertension (HTN)	1,522 (38.3)
Diabetes Mellitus (DM)	904 (22.8)
Ischemic Heart Disease (IHD)	233 (5.9)
Valve Disease	258 (6.5)
Atrial Fibrillation (AF)	203 (5.1)
Sickle Cell disease	18 (0.5)
Smoking	844 (21.3)
CKD (chronic kidney disease)	79 (2.0)
DLP (dyslipidemia)	578 (14.6)
Previous stroke	196 (4.9)
Previous TIA	90 (2.3)
Others (vasculitis, SLE, and substance abuse)	655 (16.5)
Investigations	
Thrombophilia screening total done	Positive
Homocysteine – (*N* = 869)	94 (2.4)
Antiphospholipid Screen (*N* = 1,245)	137 (3.5)
Protein C (*N* = 847)	55 (1.4)
Protein S (*N* = 836)	44 (1.1)
Thrombin mutation (*N* = 556)	31 (0.8)
Sickling for non SCD patients (*N* = 962)	16 (0.4)
Other tests	
**TEE/TTE – Positive**	**443 (18.1)**
PFO	143 (32.3)
Vegetation	48 (10.8)
Cardiac thrombus	74 (16.7)
^a^Other abnormalities	198 (44.7)
**CTA Carotid – Positive** ^b^	**2,677 (67.4)**
Carotid stenosis	356 (9.0)
Basilar stenosis/thrombosis	57 (1.4)
^c^Other stenosis	90 (2.3)
Dissection	111 (2.8)
Aneurysm	163 (4.1)
^d^Other findings	152 (3.8)
Stroke subtype	*N* = 3,971
Ischemic stroke (IS)	2,524 (63.6)
Intracerebral hemorrhage (ICH)	761 (19.2)
Cerebral venous sinus thrombosis (CVST)	413 (10.4)
Subarachnoid hemorrhage (SAH)	312 (7.9)
TOAST classification for ischemic stroke	*N* = 2,524
Large vessel disease (LVD)	554 (21.9)
Cardioembolic (CE)	466 (18.5)
Small vessel disease (SVD)	541 (21.4)
Other determined etiology (ODE)	402 (15.9)
Undetermined etiology (UDE)	561 (22.2)
Treatment	*N* = 3971
IV Thrombolysis (IVT)	337 (8.5)
Mechanical Thrombectomy (MT)	147 (3.7)
Combined (IVT + MT)	90 (2.3)
EVT (E.G. coiling, other embolization)	207 (5.2)
^e^Surgical intervention	221 (5.6)
^f^Other conservative measures	1,316 (33.1)
Anti-coagulation	801 (20.2)
Antiplatelet	2,239 (56.4)

a Apical hypokinesia/akinesia, mild LV hypo kinesis, global hypokinesia, severe ischemic cardiomyopathy, hypertensive heart disease, mild impaired LV relaxation, septal hyperkinesia, diastolic dysfunction, concentric LVH – infiltrative disease, left atrium mass, crescent shape membrane on RCC without stenosis effect, rheumatic valve disease etc.

b Positive CTA/Doppler defined as ≥50% stenosis or the presence of vascular abnormalities (aneurysm, dissection, basilar stenosis, carotid web, non-significant stenosis <50%, or plaque).

c PCA stenosis, PICA stenosis, ACA stenosis.

d Diffuse atherosclerosis, increase intima-media thickness, Moyamoya disease, parietal AVM, developmental venous anomaly, vasculitis, PCA stenosis, on-visualized right VA, complete occlusion of left vertebral artery, mild narrowing of VA, BA, duplicated AICA on right side/L pica hypo plastic, soft plaque in proximal left subclavian. Tandem occlusion of left VA, soft plaque in B/L ICA, narrowed left PCA, left cavernous ICA atherosclerosis etc.

e Clipping, Hemicraniectomy, hematoma evacuation, narrowed left PCA, left cavernous ICA atherosclerosis.

f Blood pressure control, hydration, deep vein thrombosis prophylaxis, antiemetics, exchange transfusion, hydroxyuria etc.

There was a significant younger age of onset among patients from Jordan and Sudan compared to other participating countries. There was significant male predominance among patients from Qatar with ratios of (5:1) and Oman (3:1) respectfully compared to other countries (Table 2).

**Table 2:** Distribution of mean age and sex by country.

Country (*n* = 3,971)	Age	Gender	
	Mean ±SD	Female	Male
		*n* (%)	*n* (%)
Qatar (*n* = 1,157)	38.29 ± 5.38	169 (14.6)	988 (85.4)
Iran (*n* = 901)	36.77 ± 6.77	468 (47.6)	516 (52.4)
Egypt (*n* = 554)	38.46 ± 7.52	226 (40.8)	328 (59.2)
Turkey (*n* = 516)	36.59 ± 7.65	248 (48.1)	268 (51.9)
Oman (*n* = 482)	37.81 ± 6.35	120 (24.9)	362 (75.1)
Saudi Arabia (*n* = 218)	36.78 ± 7.32	93 (42.7)	125 (57.3)
Bahrain (*n* = 121)	41.86 ± 7.43	70 (57.9)	51 (42.1)
Others (*n* = 22)	27.14 ± 8.07	7 (31.8)	15 (68.2)
*p*-value	<0.001*	<0.001*	

Hypertension (HTN) was the most common risk factor among all stroke subtypes (38%), followed by diabetes mellitus (DM) (22.4), dyslipidemia (DLP) (14.2), smoking (14.1) and 7.2% of patients had previous stroke and transient ischemic attack (Table 1).

Risk factors distribution by stroke subtypes showed that HTN, DM, DLP and smoking were the most frequent risk factors for IS and ICH while DM, HTN and smoking were common among CVST patients. Smoking and HTN were the most common in SAH (Table 3).

**Table 3:** Distribution of risk factors by stroke subtypes.

Risk factor	Stroke subtype			
	IS (*n* = 2,524)	ICH (*n* = 761)	CVST (*n* = 413)	SAH (*n* = 312)
	*n* (%)	*n* (%)	*n* (%)	*n* (%)
HTN	996 (39.5)	449 (59.0)	22 (5.3)	70 (22.4)
DM	711 (28.2)	151 (19.8)	33 (8.0)	11 (3.5)
DLP	477 (18.9)	88 (11.6)	12 (2.9)	1 (0.3)
Valve disease	215 (8.5)	24 (3.2)	17 (4.1)	2 (0.6)
Smoking	374 (14.8)	89 (11.7)	19 (4.6)	85 (27.2)
AF	187 (7.4)	9 (1.2)	6 (1.5)	1 (0.3)
IHD	205 (8.1)	22 (2.9)	5 (1.2)	3 (1.0)
Previous stroke	162 (6.4)	26 (3.4)	3 (0.7)	6 (1.9)
CKD	50 (2.0)	20 (2.6)	1 (0.2)	8 (2.6)
SCD	13 (0.5)	1 (0.1)	3 (0.7)	1 (0.3)
Previous TIA	87 (3.4)	1 (0.1)	2 (0.5)	0 (0)
Others (vasculitis, SLE, substance abuse)	338 (13.4)	111 (14.6)	143 (34.6)	82 (26.3)

IS, ischemic stroke; ICH, intracerebral hemorrhage; CVST, cerebral venous sinus thrombosis; SAH, subarachnoid hemorrhage; HTN, hypertension; DM, diabetes Mellitus; DLP, dyslipidemia; AF, atrial fibrillation; IHD, ischemic heart disease; CKD, chronic kidney disease; SCD, sickle cell disease; TIA, transit ischemic attack; SLE, systemic lupus erythematosus.

The ranking of risk factors varied between countries. For example, in Egypt, HTN [233 (42.1%)] smoking [159 (28.7%)] are the most common risk factors. Whereas in Turkey smoking [131 (25.4%)], other risk factors such vasculitis, systematic lupus erythematosus (SLE), and substance abuse [116 (22.2%)] and HTN the three most common encountered risk factors. In Oman, HTN, DM were the two most common risk factors [183 (38.0%)] and [117 (24.3%)], respectively. Previous history of transient ischemic attack (TIA) was the highest among Egyptian [63 (11.4%)], followed by Omanis [7 (1.5%)] and finally Iranian [10 (1.1%)] (Table 4).

**Table 4:** Distribution of risk factors by country.

Risk factor	Qatar (*n* = 1,157)	Iran (*n* = 901)	Egypt (*n* = 554)	Turkey (*n* = 516)	Oman (*n* = 482)	Saudi A (*n* = 218)	Bahrain (*n* = 121)	Others (*n* = 22)	*P*-value
	*n* (%)	*n* (%)	*n* (%)	*n* (%)	*n* (%)	*n* (%)	*n* (%)	*n* (%)	
HTN	661 (57.1)	249 (27.6)	233 (42.1)	86 (16.7)	183 (38.0)	62 (28.4)	45 (37.2)	3 (13.6)	<0.001*
DM	522 (45.1)	61 (6.8)	114 (20.6)	26 (5.0)	117 (24.3)	47 (21.6)	17 (14.0)	0 (0)	<0.001*
DLP	414 (35.8)	20 (2.2)	8 (1.4)	15 (2.9)	62 (12.9)	49 (22.5)	10 (8.3)	0 (0)	<0.001*
Valve disease	96 (8.3)	13 (1.4)	95 (17.1)	28 (5.4)	11 (2.3)	9 (4.1)	6 (5.0)	0 (0)	<0.001*
Smoking	287 (24.8)	175 (19.4)	159 (28.7)	131 (25.4)	49 (10.2)	29 (13.3)	12 (9.9)	2 (9.1)	<0.001*
AF	19 (1.6)	32 (3.6)	123 (22.2)	15 (2.9)	7 (1.5)	1 (0.5)	6 (5.0)	0 (0)	<0.001*
IHD	43 (3.7)	38 (4.2)	94 (17.0)	23 (4.5)	15 (3.1)	11 (5.0)	9 (7.4)	0 (0)	<0.001*
Previous stroke	62 (5.4)	64 (7.1)	34 (6.1)	11 (2.1)	21 (4.4)	4 (1.8)	0 (0)	0 (0)	<0.001*
CKD	16 (1.4)	0 (0)	11 (2.0)	12 (2.3)	24 (5.0)	11 (5.0)	5 (4.1)	0 (0)	<0.001*
SCD	0 (0)	0 (0)	2 (0.4)	0 (0)	5 (1.0)	5 (2.3)	0 (0)	6 (27.3)	<0.001*
Previous TIA	7 (0.6)	10 (1.1)	63 (11.4)	1 (0.2)	7 (1.5)	2 (0.9)	0 (0)	0 (0)	<0.001*
**Others	9 (0.8)	285 (31.6)	75 (13.5)	116 (22.2)	79 (16.4)	53 (24.3)	33 (27.3)	5 (22.7)	<0.001*

IS, ischemic stroke; ICH, intracerebral hemorrhage; CVST, cerebral venous sinus thrombosis; SAH, subarachnoid hemorrhage; HTN, hypertension; DM, diabetes Mellitus; DLP, dyslipidemia; AF, atrial fibrillation; IHD, ischemic heart disease; CKD, chronic kidney disease; SCD, sickle cell disease; TIA, transit ischemic attack; SLE, systemic lupus erythematosus.

* Statistically significant, Test: Chi-square test (Likelihood Ratio) ** Others (vasculitis, SLE, and substance abuse).

Stroke subtypes in order of frequency were ischemic stroke (IS) 63.6%, intracerebral hemorrhage (ICH) 19.2%, cerebral venous sinus thrombosis (CVST) 10.4% and subarachnoid hemorrhage (SAH) 7.9%. According to Toast classification for IS, undetermined etiology (UDE) was most common type 22.2%, followed by large vessel disease (LVD) 21.9%, small vessel disease (SVD) 21.4%, cardioembolic (CE) 18.5% and other determined etiology (ODE) 15.9% (Table 1).

**Figure 1:**
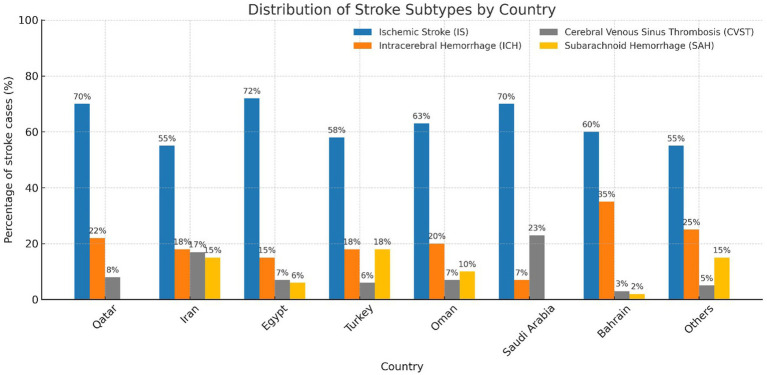
Distribution of stroke subtypes by country. Proportion of stroke cases in young adults across eight participating MENA countries, stratified by stroke subtype. IS, ischemic stroke; ICH, intracerebral hemorrhage; CVST, cerebral venous sinus thrombosis; SAH, subarachnoid hemorrhage. Percentages represent the proportion of each subtype among all stroke cases within each country.

IS was the most common stroke subtype across all the courtiers ranging from (54%) in Iran to 72% in Egypt. The highest percentage of CSVT was recorded form Saudi Arabia with 24% followed by Iran 18%. SAH was high in Turkey 20%, Jordan, Sudan 18% and Iran 14%. Whereas ICH was significantly higher as total percentage of all strokes in patients from Bahrain 34% Jordan, Sudan (27%) and Qatar (22%) (Figure 1).

### Risk factor associations with stroke subtypes

In multinomial regression, hypertension was strongly associated with ICH versus IS (aOR 2.99, 95% CI 2.33–3.83, *p* < 0.001), while younger age predicted CVST and SAH (OR per year 0.95 and 0.96, respectively, both *p* < 0.05). Conversely, diabetes (aOR 0.42 for ICH; 0.13 for SAH), dyslipidemia (aOR 0.19 for CVST), and smoking (aOR 0.37 for CVST) were significantly less frequent in these subtypes compared with IS, highlighting distinct pathophysiologic profiles across young adult stroke phenotypes. These associations remained robust after adjusting for country, though some odds ratios were inflated by sparse strata (e.g., no SAH cases from Qatar) (Supplementary Table S3).

**Figure 2:**
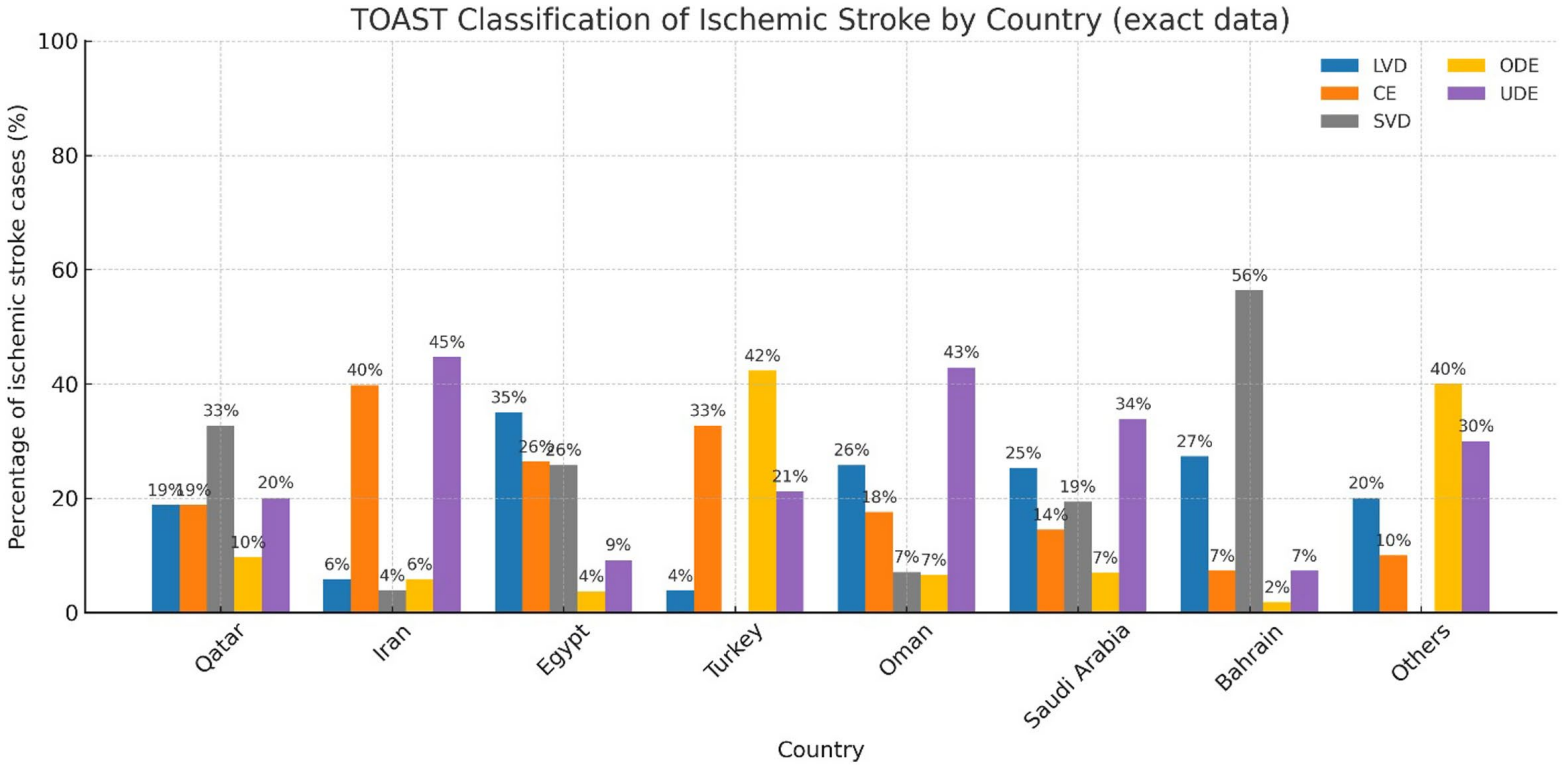
TOAST classification of ischemic stroke by country. Proportions of ischemic stroke cases among young adults in the MENA study cohort, stratified according to TOAST etiological subtypes: large-artery atherosclerosis (LVD, blue), cardioembolism (CE, orange), small-vessel disease (SVD, gray), other determined etiology (ODE, yellow-gold), and undetermined etiology (UDE, purple). Percentages represent the proportion of each subtype within the ischemic stroke cases for each country.

The Toast classification revealed the following, LVD was the most common etiology of IS in Egypt 42.5% and Bahrain 32.9%. Whereas in Turkey CE etiology was the most common cause of IS 23.6% followed by 22.8% in Egypt and 20% in Qatar. SVD was the most common cause of IS in Bahrain 39.7% followed by Qatar 32.3%, and Saudi Arabia 22.2%. ODE was the highest among Jordan and Sudan 83.3% followed by Iran 39.2% and Turkey. lastly UDE was significantly higher in Oman and Saudi Arabia patients (Figure 2).

### TOAST-risk factor associations

In multinomial regression, among ischemic strokes (*n* = 1,549; base = LVD), cardioembolism (CE) was independently associated with atrial fibrillation (aOR 7.58, 95% CI 3.13–18.5, *p* < 0.001), ischemic heart disease (aOR 5.24, 2.58–10.6, *p* < 0.001), and valvular disease (aOR 3.01, 1.63–5.53, *p* < 0.001), and with younger age (OR per year 0.97, 0.94–1.00, *p* = 0.046). Small-vessel disease (SVD) was linked to hypertension (aOR 1.80, 1.29–2.52, *p* = 0.001) and dyslipidemia (aOR 1.64, 1.14–2.38, *p* = 0.008). Diabetes was inversely associated with CE, ODE, and UDE. After adjusting for age, sex, and vascular risk factors, country of enrollment was not significantly associated with TOAST distribution (likelihood ratio test *LRT χ*^2^ = 32.8, *df* = 52, *p* = 0.983).

### Vascular imaging and thrombophilia blood test panel

All patients had either carotid doppler or cerebral CT angiogram. 32.6% of them showed negative results while 67.4% showed positive results defined as ≥50% stenosis or abnormal vascular finding, including aneurysm, dissection, carotid web, or plaque. The abnormal findings were in order of frequency, carotid stenosis (9%), aneurysm 4.1%, dissection 2.8% and basilar stenosis 1.4%. Out of 2,524 Ischemic stroke patients, 2,441 (97%) had TTE and/or TEE. 18.1% of the patients undergoing echocardiogram showed abnormalities. The abnormal findings were PFO, vegetation’s, cardiac thrombus and others. In this study, from 15 to 30% of the patients had thrombophilia blood panel tests depends on center protocol for stroke type and availability. The antiphospholipid screening tests were done in 31.4% of the patients and were positive only in 3.5% (Table 1).

### Treatment options

Treatment options delivered to all stroke subtypes varied depending on the subtypes of stroke and the etiologies. Intravenous thrombolysis therapy (IVT) was given in 8.5% of patients and Mechanical thrombectomy (MT) was performed in 3.7%. Where 2.3% received combined therapy [IVT + MT]. Anticoagulation therapy was offered in 20% of patients. EVT was performed in 5.2% of patients. Surgical intervention including clipping or hemicortectomy or hematoma evacuation was done in 5.6% of patients (Table 1).

Iran showed a significantly higher rate of IVT 30.9% compared to others, while MT was higher in Turkey 14.5. Combined therapy (IVT and MT) was significantly higher 7.8% among Egyptian patients. Anticoagulation was used in 42 and 40.7% of Egyptian and Turkish IS patients, respectively. Antiplatelets was the most common therapy in treatment of IS across all countries ranging from 58% up to 91% in Iranian patients (Supplementary Table S3).

## Discussion

The MENA region has seen notable shifts in stroke epidemiology over the past three decades. While age-standardized stroke mortality and disability-adjusted life years (DALYs) declined by 27.8 and 32%, respectively, from 1990 to 2019, the absolute number of cases has grown—driven by population expansion and aging (10, 11). Young adults, in particular, bear a disproportionate burden, with incidence rates reaching up to 40 per 100,000 person-years in some areas.

To our knowledge, this is the first multicenter study in the region assessing stroke risk factors and etiologies specifically among young adults. We included 3,971 patients from sixteen centers across nine countries. Most of the data came from high volume centers such as Qatar and Iran. This distribution likely reflects institutional stroke-center capacity and registry availability rather than true population prevalence. All centers contributed consecutive acute-stroke admissions within the study period, ensuring internal validity. Nevertheless, the over-representation of high-volume centers may limit generalizability to the entire MENA region.

While several global studies have explored young-onset stroke, most focused on a single subtype (12–16).

A marked male predominance was evident—5:1 in Qatar and 3:1 in Oman—compared to a ratio of 1.7:1 to 2.1:1 in Egypt, Saudi Arabia, and Iran (17–19). This contrasts with older populations, where the gender gap is less pronounced (20, 21). Potential explanations include higher prevalence of traditional vascular risk factors in young men (e.g., smoking, dyslipidemia, early-onset hypertension), the protective effect of estrogen in premenopausal women, and differing etiological profiles.

Cardiovascular risk factors in young adults have evolved rapidly. Hypertension and diabetes are being diagnosed earlier, and hyperlipidemia is more frequently observed than in the past (22). Meanwhile, lifestyle-related risks are growing—physical inactivity, unhealthy diets rich in sodium and processed foods, and rising use of substances such as vaping and marijuana (23).

Emerging contributors like sleep disorders, psychological stress, air pollution, and broader social determinants (e.g., income inequality, healthcare access) are gaining recognition (24). in addition, stroke subtypes more common in young adults—such as arterial dissection, patent foramen ovale, and hypercoagulable states—are being increasingly identified (25).

Together with other studies, our findings highlight a concerning rise in stroke among adults under 50, despite declining rates in older populations—suggesting that these evolving risk profiles are having real-world clinical consequences (26).

### Vascular risk factors

Across the MENA region, young stroke patients exhibit distinct vascular risk profiles. Hypertension remains the most common risk factor, though its prevalence is lower than in older populations (11, 27). Smoking is particularly frequent in countries like Egypt, Turkey, and Qatar, consistent with prior reports from North African nations such as Tunisia and Morocco (28). Dyslipidemia is notably more prevalent in Gulf countries—including Qatar, Saudi Arabia, Oman, and Bahrain—while diabetes shows an east–west gradient, being more common in Gulf states and Egypt than in Turkey and Iran (13, 29). Non-traditional contributors like vasculitis, SLE, substance use, and hormonal factors (e.g., oral contraceptives) vary by setting. Our regression findings deepen earlier descriptive work by quantifying how risk factors guide differing stroke subtypes. In line with population-based studies such as Putaala et al.’s Helsinki Young Stroke Registry (where hypertension, dyslipidemia, and smoking were dominant modifiable risks) (30), we found hypertension to be the dominant driver of both ICH and small-vessel disease. Cardioembolism in our cohort was powerfully predicted by atrial fibrillation, ischemic heart disease, and valvular disease, echoing findings in Western cohorts where structural and rhythm-related cardiac conditions are strong embolic sources.

### Stroke subtypes

#### Ischemic stroke

Ischemic Stroke (IS) is the dominant stroke subtype in the region, comprising 63.6% of cases. Subtype distribution varies widely. Large vessel disease (LVD) is frequent in Egypt, Bahrain, and Oman but remains lower than reported in Western populations (31). Small vessel disease is prominent in Qatar and Saudi Arabia, in line with the burden of metabolic syndrome (32). Cardioembolic strokes are common in Turkey and Egypt, reflecting the prevalence of rheumatic heart disease (5). Cryptogenic strokes remain high, likely due to gaps in diagnostic resources (12).

#### Intracerebral hemorrhage

In individuals under 45, ICH affects about 7 per 100,000 annually—and up to 14 per 100,000 among young Black males (33). It’s more prevalent in males and in ethnic minorities (34). Intraparenchymal hemorrhage accounts for approximately 20% of young strokes (35), aligning with our cohort. Rates are higher in Asian populations (36). Causes differ from older adults and include hypertension, coagulopathies, arteriovenous malformations, drug abuse (e.g., cocaine), Moyamoya disease, and hereditary forms of cerebral amyloid angiopathy (37–40). In MENA, sickle cell anemia is a notable contributor to Moyamoya-related ICH. Early hypertension detection and control may mitigate spontaneous ICH risk in this age group.

#### Cerebral venous sinus thrombosis

Cerebral Venous Sinus Thrombosis (CVST) accounts for approximately 0.5% of all strokes globally (41), but 10–15% of young strokes (42, 43). In our cohort, CVST made up 10.4% of young adult strokes—higher than many Western reports but lower than rates in India (up to 16.3%) (44, 45). Anticoagulation remains the mainstay treatment (41), used in 80.3% of cases. Antiplatelets (3.5%), thrombectomy (1%), and other interventions (13.6%) were also reported. While global recurrence risk is 2–3% (46) our observed rate was 0.05%, likely due to limited follow-up. CVST was more frequent in young women, often associated with pregnancy in Saudi Arabia and Iran (47).

#### Subarachnoid hemorrhage

Subarachnoid Hemorrhage (SAH) appears less common in the MENA region, possibly due to underreporting, with estimated incidence around 1.5–2.5 per 100,000 (48). In our cohort, SAH represented 7.9% of cases. Prior studies suggest a younger age of onset in MENA, this was supported by our multimodal regression analysis showed that younger age was independently associated with CVST and SAH, highlighting a distinct etiologic signature for these subtypes, consistent with reports from South Asian and European young stroke series (49, 50). This is potentially related to higher rates of hypertension and smoking (51). In a report from the region, SAH patients, 27.2% were found to be smokers and 50.1% had hypertension (52). Delays in aneurysm treatment are common due to health system limitations (53). Endovascular techniques were preferred over clipping in our cohort (40 of 52 cases).

#### Regional variation in TOAST subtypes

In our cohort, small-vessel disease (SVD, 25%) emerged as the leading TOAST subtype, consistent with the high prevalence of hypertension and dyslipidemia among young adults in the MENA region. Cardioembolism (CE, 20%) was most strongly predicted by atrial fibrillation, ischemic heart disease, and valvular pathology, underscoring the role of structural and electrical cardiac substrates even in this younger age group. Notably, AF—typically uncommon in Asian young-stroke cohorts (30, 54). emerged with a strong signal in our data, likely reflecting both rigorous cardiac work-up and the demographic mix of populations with higher AF susceptibility. In addition, premature coronary artery disease and left-ventricular wall-motion abnormalities are increasingly recognized as important competing embolic substrates in MENA populations are often categorized as cryptogenic and are independently associated with major adverse cardiac events (MACE).

Cervical artery dissection (CeAD), which is often cited as accounting for ~10–20% of young ischemic strokes globally (55), was detected in only 2.8% of our cohort. This is consistent with regional registry data, for example, in central Iran only 1.4% of ischemic strokes were attributed to cervical dissection (56). Another study showed 6.4% prevalence among young Saudi stroke (57). While the low proportion may partly reflect ascertainment/diagnostic differences, the alignment with regional data suggests that true etiological variation cannot be ignored. In addition, Premature coronary artery disease and left-ventricular wall-motion abnormalities are emerging as key embolic substrates in MENA populations, frequently classified under cryptogenic stroke, and are independently linked to higher risk of major adverse cardiovascular events (MACE) (58).

Large-vessel disease (LVD, 21%) was disproportionately represented in North African centers, where extracranial carotid atherosclerosis has historically been more common; for example, Egyptian cohorts have reported ≥70% carotid stenosis in up to 23.8% of stroke patients, though intracranial disease still predominates on advanced imaging (59, 60). More broadly, higher rates of intracranial atherosclerosis have been documented in Asian and Middle Eastern populations compared with the extracranial burden more typical of European and North American cohorts (61), placing North Africa in an intermediate position within the MENA region.

When contrasted with international cohorts (62), our distribution aligns more closely with Asian registries, where SVD predominates (30, 54), than with Western series, where cryptogenic etiologies are most common. By contrast, undetermined etiology (UDE, 25%) remained frequent in our cohort, particularly in Gulf states. Importantly, this predominance cannot be ascribed to limited access to diagnostics, as advanced cardiac and vascular imaging is readily available in Qatar, Saudi Arabia, and the UAE. Rather, it may reflect the younger age structure of affected patients, where traditional risk factors are less penetrant, the inherently cryptogenic nature of some strokes (e.g., patent foramen ovale–related embolism), and incomplete detection of transient arrhythmias despite modern monitoring.

These inter-regional contrasts raise questions about the interplay between genetics, vascular biology, and environmental exposures. The persistence of these subtype differences, even in settings with excellent diagnostic capacity, suggests that regional young-stroke epidemiology is shaped less by healthcare infrastructure and more by underlying biology and demographics.

### Regional challenges

Diagnostic capabilities vary widely, with Gulf states having greater access to advanced imaging (63). Limited availability of genetic testing further hinders the recognition of hereditary stroke syndromes (64). Rehabilitation infrastructure is also uneven, with poorer outcomes in countries lacking comprehensive post-stroke care.

### Treatment options

Guidelines for managing stroke in young adults often mirror those for older adults, despite significant differences. Young adults are underrepresented in trials, and dedicated subgroup analyses are rare. Sickle cell disease (a major global contributor to pediatric stroke) responds well to hydroxyurea. Genetic therapies are evolving for rare monogenic stroke disorders like adenosine deaminase 2 deficiency, hemophilia, and Fabry disease. In cryptogenic stroke, careful assessment for patent foramen ovale (PFO) is essential. Though PFO is present in ~25% of the population (65), it can facilitate paradoxical embolism (66).

### Recommendations

To address the growing burden of stroke in young adults across the MENA region, we propose:

- *Regional Stroke Registries*: Establish standardized data collection platforms to support research, benchmarking, and policy development.
- *Unified Diagnostic Protocols*: Standardize workups for young patients to reduce the rate of cryptogenic strokes, including screening for rare causes like dissection or thrombophilia.
- *Public Health Campaigns*: Target youth with education on smoking cessation, nutrition, exercise, and under recognized risk factors (e.g., sleep disorders, stress).
- *Early Risk Factor Screening*: Empower primary care systems to detect and manage hypertension, diabetes, and dyslipidemia earlier.
- *Improved Rehabilitation Access*: Invest in multidisciplinary rehab services, especially for young survivors facing long-term disability.
- *Regionally Relevant Research*: Encourage collaboration and support local investigators to study region-specific risk factors, genetics, and care models.

### Limitations

This study has several limitations that should be acknowledged. First, although data were collected using a unified template, diagnostic investigations and TOAST classification were performed locally, introducing potential inter-site variability. Second, country representation was not proportional to national stroke volumes, which may limit regional generalizability. Third, clinical outcomes such as disability at discharge or 3-month mortality were unavailable across several centers, precluding analysis of predictors of prognosis. Fourth, some etiologic investigations (e.g., genetic and thrombophilia testing) were performed selectively, possibly leading to underestimation of rare causes. Finally, despite these constraints, the study remains the largest coordinated effort to characterize young-adult stroke in the MENA region and provides valuable groundwork for future prospective, outcome-based research.

## Conclusion

This study represents the first regionally coordinated effort to explore stroke in young adults across the MENA region. Our findings reveal not only a significant disease burden but also striking heterogeneity in patient demographics, risk profiles, and healthcare responses. A strong male predominance, the emergence of non-traditional risk factors, and ongoing diagnostic gaps highlight the complexity of this growing public health issue. Nonetheless, this complexity presents a timely opportunity for innovation, collaboration, and targeted action.

## Data Availability

The original contributions presented in the study are included in the article/Supplementary material, further inquiries can be directed to the corresponding author.

## Group member of Oman Stroke Society

www.omanstrokesociety.com.

## Glossary

AF: Atrial fibrillation
ACA: Anterior cerebral artery
ASA: Atrial septal aneurysm
BA: Basilar artery
CE: Cardioembolic (TOAST subtype)
CeAD: Cervical artery dissection
CI: Confidence interval
CKD: Chronic kidney disease
CTA: Computed tomographic angiography
CT/CTV: Computed tomography/CT venography
CSVT: Cerebral (sinus) venous thrombosis
DALYs: Disability-adjusted life year(s)
DM: Diabetes mellitus
DLP: Dyslipidemia
ECG: Electrocardiogram
EVT: Endovascular treatment
HTN: Hypertension
HR: Hazard Ratio
ICH: Intracerebral hemorrhage
IHD: Ischemic heart disease
IS: Ischemic stroke
IVT: Intravenous thrombolysis
LRT: Likelihood-ratio test
LVD: Large vessel disease (TOAST subtype)
LVO: Large vessel occlusion
NIHSS: National Institutes of Health Stroke Scale
MACE: Major adverse cardiovascular events
MCA: Middle cerebral artery
MENA: Middle East and North Africa
MRI/MRV: Magnetic resonance imaging/MR venography
MT: Mechanical thrombectomy
mRS: Modified Rankin Scale
ODE: Other determined etiology (TOAST subtype)
PCA: Posterior cerebral artery
PFO: Patent foramen ovale
PICA: Posterior inferior cerebellar artery
SCD: Sickle cell disease
SD: Standard deviation
SLE: Systemic lupus erythematosus
SPSS: Statistical Package for the Social Sciences
SVD: Small vessel disease (TOAST subtype)
SAH: Subarachnoid hemorrhage
TIA: Transient ischemic attack
TTE: Transthoracic echocardiogram
TEE: Transesophageal echocardiogram
TOAST: Trial of Org 10,172 in Acute Stroke Treatment classification
UDE: Undetermined etiology (TOAST subtype)
VA: Vertebral artery
VIF: Variance inflation factor
WHO: World Health Organization
aOR: Adjusted odds ratio
