# Supplementary material for: Stroke in young adults in the Middle East and North Africa region: What is the difference from elsewhere? A report from sixteen centers experiences
Source: Front Neurol. 2026 Apr 10;16:1653599. doi: 10.3389/fneur.2025.1653599 (PMC13105874; doi:10.3389/fneur.2025.1653599)
Supplement: Supplementary file 1 [file Table_1.docx]

**Table S1. Multinomial logistic regression for stroke subtypes (Reference category = Ischemic Stroke, IS). Models adjusted for age, sex, and country.**

| **Outcome vs IS (Ref)** | **Predictor** | **aOR** | **95% CI** | **p-value** |  |
| --- | --- | --- | --- | --- | --- |
| ICH vs IS | Hypertension | 2.99 | 2.33–3.83 | <0.001 |  |
| CVST vs IS | Age (per year) | 0.95 | — | <0.05 |  |
| SAH vs IS | Age (per year) | 0.96 | — | <0.05 |  |
| ICH vs IS | Diabetes | 0.42 | — | <0.05 |  |
| SAH vs IS | Diabetes | 0.13 | — | <0.05 |  |
| CVST vs IS | Dyslipidemia | 0.19 | — | <0.05 |  |
| CVST vs IS | Smoking | 0.37 | — | <0.05 |  |
| Notes: Associations remained after adjusting for country. | | | | | |
