# Supplementary material for: Stroke in young adults in the Middle East and North Africa region: What is the difference from elsewhere? A report from sixteen centers experiences
Source: Front Neurol. 2026 Apr 10;16:1653599. doi: 10.3389/fneur.2025.1653599 (PMC13105874; doi:10.3389/fneur.2025.1653599)
Supplement: Supplementary file 2 [file Table_2.docx]

**Table S2. Multinomial logistic regression for TOAST classification among ischemic stroke (Reference category = Large Vessel Disease, LVD). Models adjusted for age, sex, and country.**

| **TOAST outcome vs LVD (Ref)** | **Predictor** | **aOR** | **95% CI** | **p-value** |  |
| --- | --- | --- | --- | --- | --- |
| CE vs LVD | Atrial fibrillation | 7.58 | 3.13–18.5 | <0.001 |  |
| CE vs LVD | Ischemic heart disease | 5.24 | 2.58–10.6 | <0.001 |  |
| CE vs LVD | Valvular disease | 3.01 | 1.63–5.53 | <0.001 |  |
| CE vs LVD | Age (per year) | 0.97 | 0.94–1.00 | 0.046 |  |
| SVD vs LVD | Hypertension | 1.80 | 1.29–2.52 | 0.001 |  |
| SVD vs LVD | Dyslipidemia | 1.64 | 1.14–2.38 | 0.008 |  |
| CE/ODE/UDE vs LVD | Diabetes | Inverse | — | <0.05 |  |
