# Supplementary material for: Stroke in young adults in the Middle East and North Africa region: What is the difference from elsewhere? A report from sixteen centers experiences
Source: Front Neurol. 2026 Apr 10;16:1653599. doi: 10.3389/fneur.2025.1653599 (PMC13105874; doi:10.3389/fneur.2025.1653599)
Supplement: Supplementary file 3 [file Table_3.docx]

***Table S3: Treatment received for IS by Country***

| **Country (no. of IS)** | **Treatment received for IS** | | | | | |
| --- | --- | --- | --- | --- | --- | --- |
|  | **IVT** | **MT** | **Combined (IV+MT)** | **Anti-coagulation** | **Antiplatelet** | **Other conservative measures** |
|  | n (%) | n (%) | n (%) | n (%) | n (%) | n (%) |
| Qatar (n= 805) | 64 (8.0) | 35 (4.3) | 22 (2.7) | 95 (11.8) | 541 (67.2) | 526 (65.3) |
| Iran (n= 482) | 149 (30.9) | 2 (0.4) | 1 (0.2) | 43 (8.9) | 440 (91.3) | 5 (1.0) |
| Egypt (n= 400) | 50 (12.5) | 29 (7.2) | 31 (7.8) | 168 (42.0) | 319 (79.8) | 19 (4.8) |
| Turkey (n= 297) | 21 (7.1) | 43 (14.5) | 17 (5.7) | 121 (40.7) | 196 (66.0) | 18 (6.1) |
| Oman (n= 302) | 18 (6.0) | 1 (0.3) | 4 (1.3) | 15 (5.0) | 255 (84.4) | 45 (14.9) |
| Saudi Arabia (n= 153) | 5 (3.3) | 11 (7.2) | 4 (2.6) | 34 (22.2) | 123 (80.4) | 0 (0) |
| Bahrain (n= 73) | 0 (0) | 0 (0) | 0 (0) | 17 (23.3) | 62 (84.9) | 0 (0) |
| Others (n= 22) | 0 (0) | 1 (8.3) | 0 (0) | 5 (41.7) | 7 (58.3) | 7 (58.3) |
| IVT: Intravenous thrombolysis, MT: Mechanical thrombectomy, IS: ischemic stroke | | | | | | |
